# Supplementary material for: Synapsin 2a tetramerisation selectively controls the presynaptic nanoscale organisation of reserve synaptic vesicles
Source: Nat Commun. 2024 Mar 12;15:2217. doi: 10.1038/s41467-024-46256-1 (PMC10933366; doi:10.1038/s41467-024-46256-1)
Supplement: Supplementary file 1 — Supplementary Information [file 41467_2024_46256_MOESM1_ESM.pdf]

Supplementary Figures:

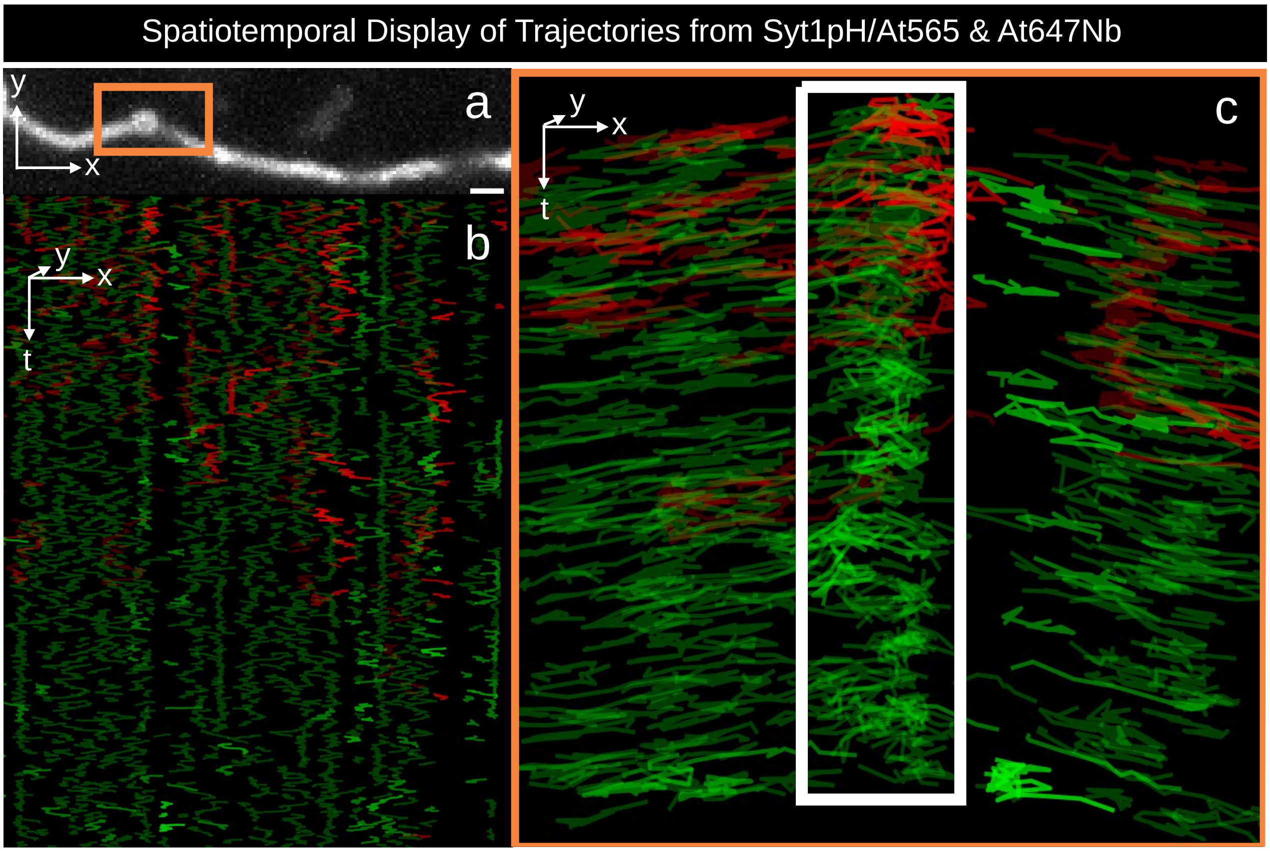

Supplementary Figure 1. Spatiotemporal matrix of SV trajectories clustering in the presynapses versus axonal segments.

Spatiotemporal display of trajectories from Synaptotagmin1-pHluorin (Syt1pH)-bound anti-green fluorescent protein (GFP) Atto 647N nanobodies (At647Nb; recycling pool; 10 min chase; green) and anti-GFP Atto 565 nanobodies (At565Nb; reserve pool; 48 h chase; red). (**a**) Epifluorescence image of a neuronal segment expressing Syt1pH acquired before stimulation, with a presynapse and flanking axonal segments highlighted with an orange box. (**b**) A 3D [x,y,t] projection of trajectories rotated such that the 'temporal columns' of trajectories at defined regions of the plasma membrane are apparent. (**c**) Magnified image of the highlighted orange box in (**a**). Trajectories in the presynapse (white box) display a higher density of spatially confined trajectories than the flanking axonal regions. Scale bar 1 µm.

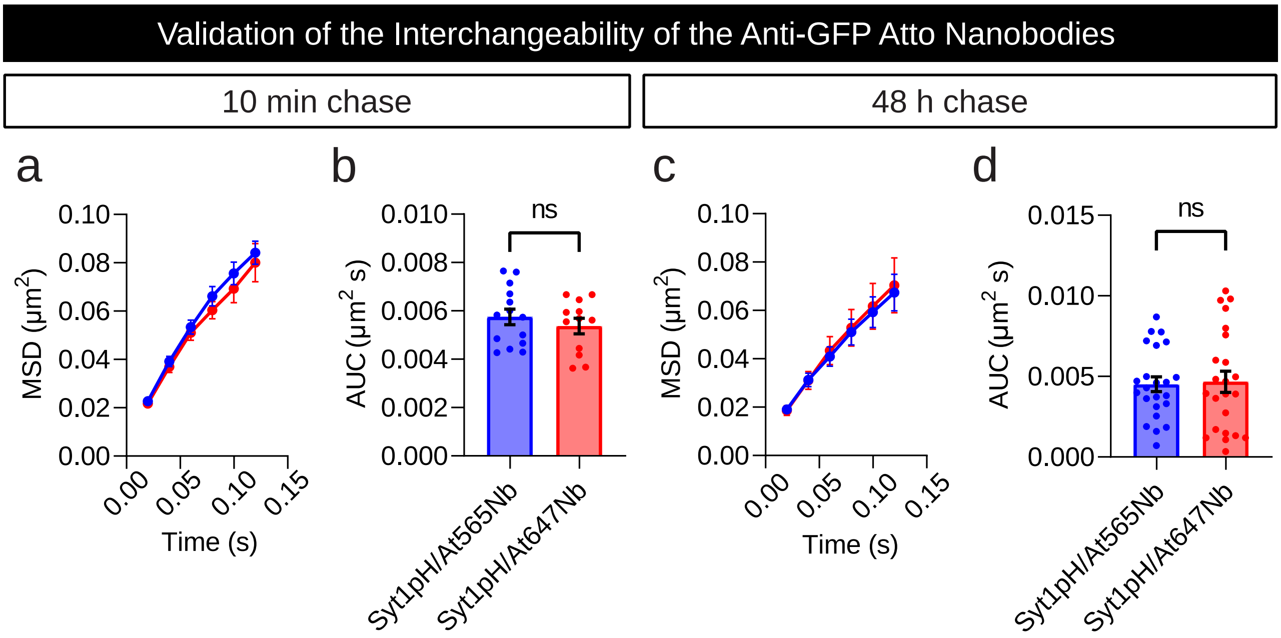

Supplementary Figure 2. Validation of the interchangeability of the anti-GFP Atto nanobodies used for DsdTIM.

Hippocampal neurons expressing Synaptotagmin1–pHluorin (Syt1pH) were stimulated with high K^+ medium containing both anti-green fluorescent protein (GFP) Atto 565-tagged nanobodies (At565Nb; blue) and anti-GFP Atto 647N-tagged nanobodies (At647Nb; red). Following stimulation, the excess nanobodies were washed off, and the neurons were chased for either (**a, b**) 10 min or (**c, d**) 48 h before imaging in a low K^+ imaging buffer. (**a, c**) Average mean square displacement (MSD; μm^2) and corresponding (**b, d**) area under the MSD curve (AUC; μm^{2}s) of the trajectories generated from either Syt1pH/At565Nb (blue) or Syt1pH/At647Nb (red) in the presynaptic compartment. Data are displayed as mean ± SEM. Values were obtained from n = 14 presynapses (Syt1pH/At565Nb) and n = 12 presynapses (Syt1pH/At647Nb) in (**a, b**) and from n = 23 presynapses (Syt1pH/At565Nb and Syt1pH/At647Nb) in (**c, d**). Data was obtained from 1 independent neuronal culture. Statistical comparisons were performed using the unpaired two-tailed Student's *t*-test in (**b**) and (**d**).

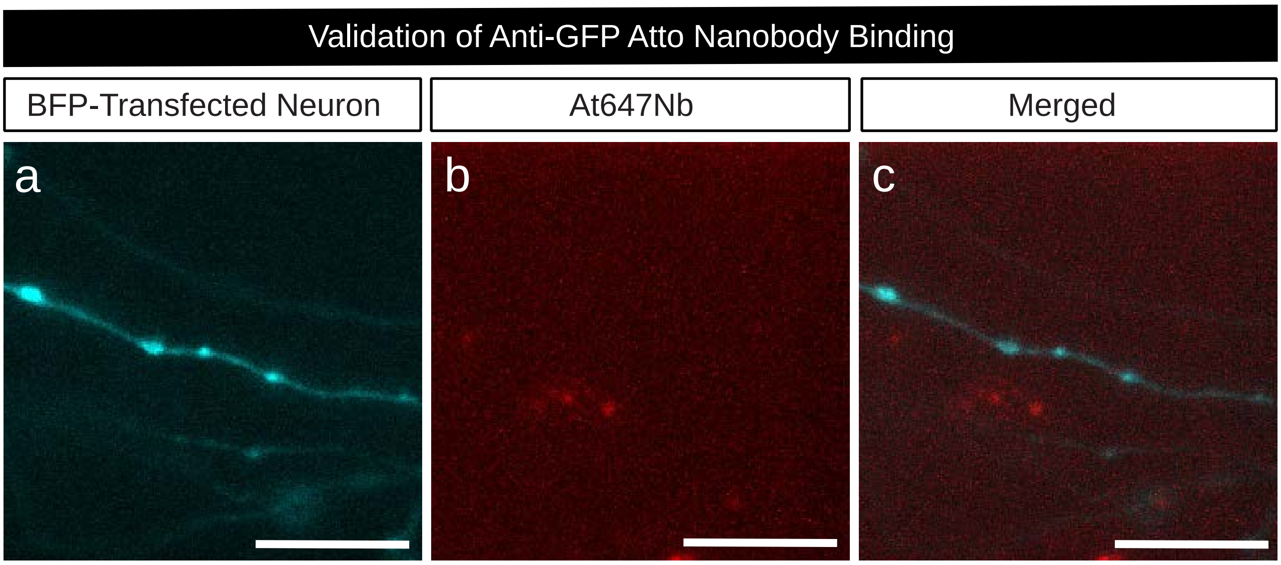

Supplementary Figure 3. Validation of anti-GFP Atto nanobody specific binding to pHluorin.

Representative image of a hippocampal neuron (**a**) transfected with blue-fluorescent protein (BFP; cyan). (**b**) Max intensity projection of the same region in (**a**) after being stimulated for 5 minutes (high K^+ buffer containing anti-green fluorescent protein (GFP) Atto 647N-tagged nanobodies; At647Nb), the excess nanobodies washed off, and chased for 10 minutes to allow for internalisation of any bound nanobody (red). (**c**) Merged image. Scale bar 10 µm (**a-c**). Data was obtained from 1 independent neuronal culture.

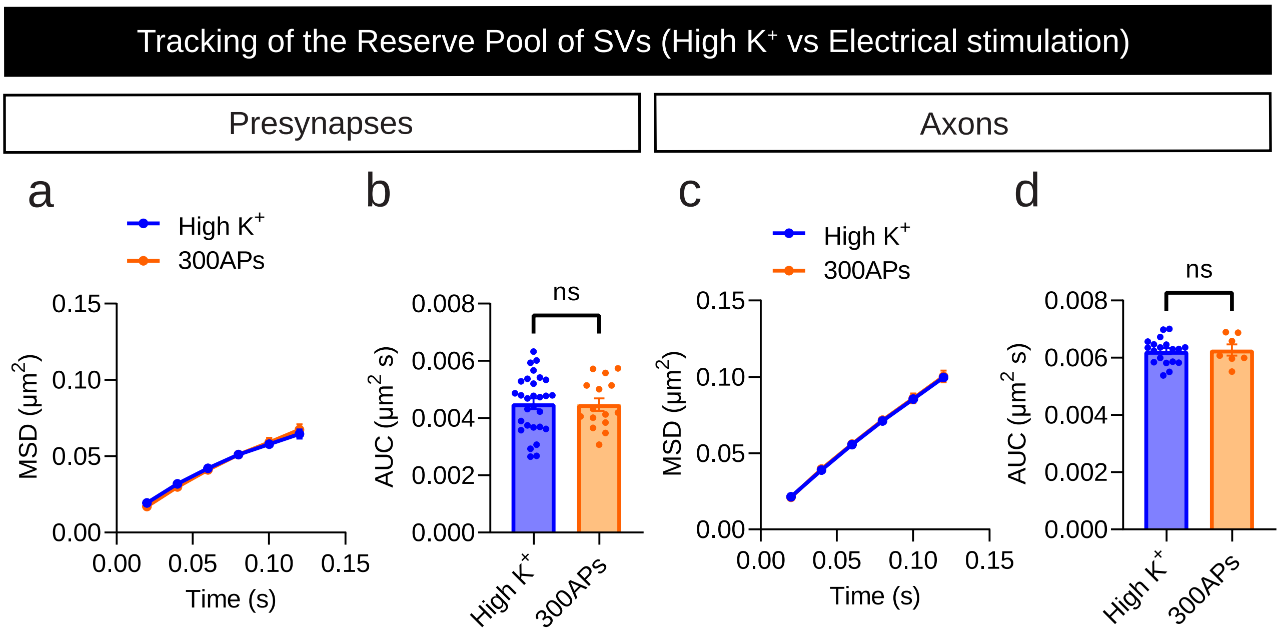

Supplementary Figure 4. Comparison of reserve SV mobility 48 hours after being labelled with either high K^+ or electrical field stimulation.

(**a, c**) Average mean square displacement (MSD; μm^2) of reserve synaptic vesicles (SVs) labelled using either a high K^+ buffer (blue) or train of 300 action potentials (APs; 50 Hz for 6 s; orange), within the (**a**) presynapses and (**c**) axons. (**b, d)** Area under the MSD curve (AUC; $\mu m^2 s$) for (**b**) presynapses and (**d**) axons. Data are displayed as mean ± SEM. Values were obtained from n = 29 presynapses (high K^+) and n = 15 presynapses (300 APs) in (**a, b**); from n = 19 axons (high K^+) and n = 7 axons (300 APs) in (**c, d**). Data was obtained from 1 independent neuronal culture. Statistical comparisons were performed using the unpaired two-tailed Student's *t*-test in (**b**) and (**d**).

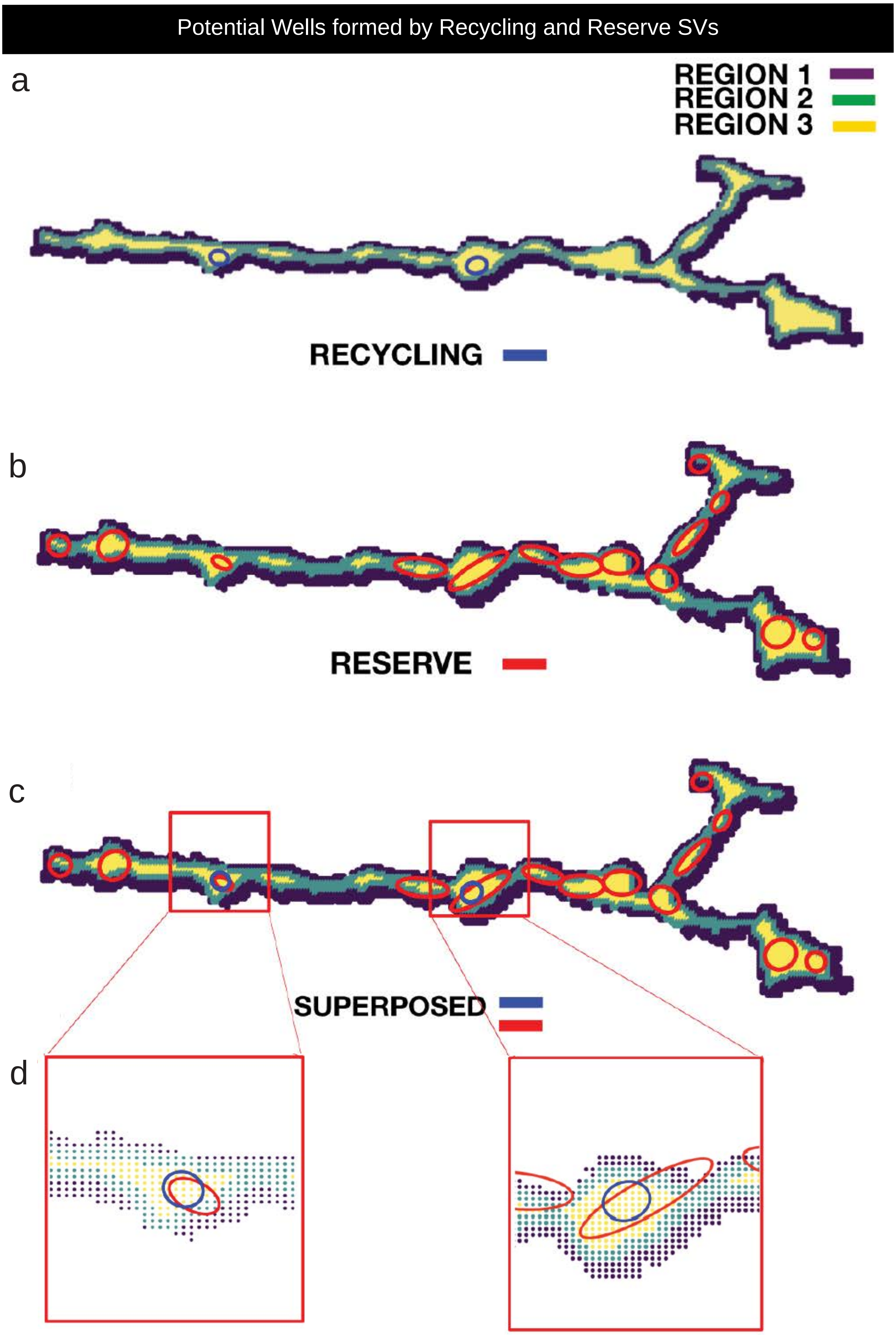

Supplementary Figure 5. High-density regions displaying potential recycling and reserve SV wells.

When quantifying high density synaptic vesicle (SV) trajectory regions, the neuron was subdivided into three sub-regions: region 1 (R1; purple) is located near the boundary, region 2 (R2, green) is the intermediate and region 3 (R3; yellow) is located close to the centre of the axon or presynapse. (**a**) Potential wells associated with high-density regions of recycling SVs (blue ellipses). (**b**) Potential wells associated with high-density regions of reserve SVs (red ellipses). (**c**) Superposition of the potential wells from the two types of SVs in the presynapse. (**d**) Magnified regions of interest to emphasize the overlapping of the different potential wells.
