# Supplementary material for: Synapsin 2a tetramerisation selectively controls the presynaptic nanoscale organisation of reserve synaptic vesicles
Source: Nat Commun. 2024 Mar 12;15:2217. doi: 10.1038/s41467-024-46256-1 (PMC10933366; doi:10.1038/s41467-024-46256-1)
Supplement: Supplementary file 2 — Reporting Summary [file 41467_2024_46256_MOESM2_ESM.pdf]

nature portfolio

Corresponding author(s): Frederic A. Meunier

Last updated by author(s): 21 February 2024

Reporting Summary

Nature Portfolio wishes to improve the reproducibility of the work that we publish. This form provides structure for consistency and transparency in reporting. For further information on Nature Portfolio policies, see our Editorial Policies and the Editorial Policy Checklist.

Statistics

For all statistical analyses, confirm that the following items are present in the figure legend, table legend, main text, or Methods section.

n/a	Confirmed	
☐	☒	The exact sample size (*n*) for each experimental group/condition, given as a discrete number and unit of measurement
☐	☒	A statement on whether measurements were taken from distinct samples or whether the same sample was measured repeatedly
☐	☒	The statistical test(s) used AND whether they are one- or two-sided<br>*Only common tests should be described solely by name; describe more complex techniques in the Methods section.*
☒	☐	A description of all covariates tested
☐	☒	A description of any assumptions or corrections, such as tests of normality and adjustment for multiple comparisons
☐	☒	A full description of the statistical parameters including central tendency (e.g. means) or other basic estimates (e.g. regression coefficient) AND variation (e.g. standard deviation) or associated estimates of uncertainty (e.g. confidence intervals)
☐	☒	For null hypothesis testing, the test statistic (e.g. *F*, *t*, *r*) with confidence intervals, effect sizes, degrees of freedom and *P* value noted<br>*Give P values as exact values whenever suitable.*
☑	☐	For Bayesian analysis, information on the choice of priors and Markov chain Monte Carlo settings
☒	☐	For hierarchical and complex designs, identification of the appropriate level for tests and full reporting of outcomes
☐	☒	Estimates of effect sizes (e.g. Cohen's *d*, Pearson's *r*), indicating how they were calculated

Our web collection on statistics for biologists contains articles on many of the points above.

Software and code

Policy information about availability of computer code

Data collection	Super-resolution imaging was acquired using Metamorph software (version 7.7.8, Molecular Devices, CA, USA), as detailed in the method section.
Data analysis	FIJI-Image J (version 2.0.0-rc-04Tll:30:09+0000), MetaMorph (Molecular Devices), PALM-Tracer (version 2.1.0.28228), GraphPadPrism software {Graph Pad Software, Inc), Microsoft Excel (version 16.62). Computer codes to analyze the data have been described in the work by Wallis et al. 2023 Nature Communications. Full details about how the code can be accessed is in that paper.

For manuscripts utilizing custom algorithms or software that are central to the research but not yet described in published literature, software must be made available to editors and reviewers. We strongly encourage code deposition in a community repository (e.g. GitHub). See the Nature Portfolio guidelines for submitting code & software for further information.

Data

Policy information about availability of data

All manuscripts must include a data availability statement. This statement should provide the following information, where applicable:

- Accession codes, unique identifiers, or web links for publicly available datasets
- A description of any restrictions on data availability
- For clinical datasets or third party data, please ensure that the statement adheres to our policy

Single-particle trajectory data generated in this study are available for download from the publicly accessible institutional data repository of The University of Queensland (UQ eSpace) 10.48610/be3832e.
Further information and request for reagents and resources should be directed to the corresponding author.
Source data are provided with this paper.

Research involving human participants, their data, or biological material

Policy information about studies with human participants or human data. See also policy information about sex, gender (identity/presentation), and sexual orientation and race, ethnicity and racism.

Reporting on sex and gender	N/A
Reporting on race, ethnicity, or other socially relevant groupings	N/A
Population characteristics	N/A
Recruitment	N/A
Ethics oversight	N/A

Note that full information on the approval of the study protocol must also be provided in the manuscript.

Field-specific reporting

Please select the one below that is the best fit for your research. If you are not sure, read the appropriate sections before making your selection.

☒ Life sciences ☐ Behavioural & social sciences ☐ Ecological, evolutionary & environmental sciences

For a reference copy of the document with all sections, see nature.com/documents/nr-reporting-summary-flat.pdf

Life sciences study design

All studies must disclose on these points even when the disclosure is negative.

Sample size	No statistical methods were used to predetermine sample sizes because our sample sizes were estimated based on those reported in similar previous publications (Joensuu et al. J Cell Biol. 2016 Oct 24;215(2):277-292; Padmanabhan et al., 2019, eLife 8:e45040; Martinez-Marmol et al., Mol Psychiatry. 2023 Feb;28(2):946-962). All the results for each condition are obtained from 2 to 4 independent neuronal dissections, pooling over 5 embryos per dissection. Respective n values are shown in figure captions.
Data exclusions	Outliers were automatically identified based on their position at over two standard deviations from the mean, using the custom-made Python script Outlier Wrangler (Wallis et al. 2023 Nature Communications). Outliers were automatically excluded from the dataset, as stated in the "Statistical analysis" section from the "Methods". Exclusion criteria was pre-established.
Replication	Neurons were collected from at least two independent experiments, and each experiment was derived from pooling neurons from over 5 dissected embryos. All replication attempts were successful.
Randomization	Analyzed neurons were selected randomly from the culture dishes. Neurons were obtained from mixing the brains of over 5 dissected embryos (numbers vary depending on the pregnancies, as all embryos from the same pregnant mouse were used). The same number of dissected neurons were plated into dishes. Plating is considered random as it is impossible to know the origin of each neuron plated (to what embryo correspond). The pool of dishes generated are mixed and randomly divided into different treatments. The assignment

	of treatments is performed by simple randomization using Excel.
Blinding	Our experiments were not performed blind. However, the data within the experiments was re-analyzed independently by different researchers. We also used computer-based single-molecule automatic detection and tracking to minimize bias. Detection, tracking and analysis conditions were maintained constant between experimental conditions.

Reporting for specific materials, systems and methods

We require information from authors about some types of materials, experimental systems and methods used in many studies. Here, indicate whether each material, system or method listed is relevant to your study. If you are not sure if a list item applies to your research, read the appropriate section before selecting a response.

Materials & experimental systems

n/a	Involved in the study	
☐	☒	Antibodies
☑	☐	Eukaryotic cell lines
☒	☐	Palaeontology and archaeology
☐	☒	Animals and other organisms
☒	☐	Clinical data
☒	☐	Dual use research of concern
☒	☐	Plants

Methods

n/a	Involved in the study	
☒	☐	ChIP-seq
☒	☐	Flow cytometry
☒	☐	MRI-based neuroimaging

Antibodies

Antibodies used	Anti-GFP nanobodies tagged with Atto647N (200 or 400pM, GFP sdAb - FluoTag-Q, #N0301-AF647-L, Synaptic Systems) or Atto565 (200 or 400pM, GFP sdAb - FluoTag-Q, #N0301-At565-L, Synaptic Systems,) were used in this study.
Validation	Nanobodies were validated by the manufacturers as indicated on the manufacturer's website (see below):<br>https://sysy.com/product/N0301-AF647-L<br>https://www.labome.com/product/Synaptic-Systems/N0301-At565-L.html

Eukaryotic cell lines

Policy information about cell lines and Sex and Gender in Research

Cell line source(s)	N/A
Authentication	N/A
Mycoplasma contamination	N/A
Commonly misidentified lines (See ICLAC register)	N/A

Animals and other research organisms

Policy information about studies involving animals; ARRIVE guidelines recommended for reporting animal research, and Sex and Gender in Research

Laboratory animals

-Wild type mice: C57BL/6 strain
-Synapsin triple (1-3) knock out mice (SynTKO; B6;129-Syn2^{tm1Pggd} Syn3^{tm1Pggd} Syn1t^{m1Pggd}/Mmjax) mice (generated in Gitler *et al.*, 2004; gift from Prof George Augustine)

Age adults used for mating: 5-12 weeks old. Experiments were performed on hippocampal neurons isolated from WT embryos (E16) and SynTKO P0 pups (no sex differentiation).

For all mice:
Cage/tank/housing system (type and dimensions): OptiMice standard caging system: 34.3cm L, 29.2cm W, 15.5cm H.
Cage floor area: 484cm².
Food: Mouse cubes (SF00-100)(Autoclaved).
Bedding: Pura Chip Aspen Fine Sani Chips- Biological Associates (Autoclaved).
Nesting material: Tissue and Enviro-Dri (Autoclaved).
Additional nesting/home cage enrichment: Red or yellow house/tunnel or cardboard house/tunnel.
Temperature and humidity parameters: 18°c - 24°c (30%-70% RH).
Lighting (type, schedule and intensity): 12 L : 12 D 80% intensity.

Wild animals	No wild animals were used in the study.
Reporting on sex	Data were collected from hippocampal neurons isolated from embryonic or post-natal pups of mixed gender
Field-collected samples	No field collected samples were used in the study.
Ethics oversight	All experimental procedures using animals were conducted under the guidelines of the Australian Code of Practice for the Care and Use of Animals for Scientific purposes and were approved by The University of Queensland (UQ) Animal Ethics Committee (2020/AE000439, 2020/AE000379 and 2023/AE000169) and Nanyang Technological University Institutional Animal Care and Use Committee (NTU-IACUC; described in the animal use protocol (AUP) A18095 / A21020).

Note that full information on the approval of the study protocol must also be provided in the manuscript.

Plants

Seed stocks	N/A
Novel plant genotypes	N/A
Authentication	N/A
